# Prognosis of septic cavernous sinus thrombosis remarkably improved: a case series of 12 patients and literature review

**DOI:** 10.1007/s00405-018-5062-9

**Published:** 2018-07-11

**Authors:** Nicolien A. van der Poel, Maarten P. Mourits, Maartje M. L. de Win, Jonathan M. Coutinho, Frederik G. Dikkers

**Affiliations:** 1 Department of Otorhinolaryngology, Academic Medical Center, University of Amsterdam, Meibergdreef 9, 1105 AZ Amsterdam, The Netherlands; 2 Department of Ophthalmology, Academic Medical Center, University of Amsterdam, Amsterdam, The Netherlands; 3 Department of Radiology and Nuclear Medicine, Academic Medical Center, University of Amsterdam, Amsterdam, The Netherlands; 4 Department of Neurology, Academic Medical Center, University of Amsterdam, Amsterdam, The Netherlands

**Keywords:** Cavernous sinus thrombosis, Anticoagulants, Orbital cellulitis, Sinusitis

## Abstract

**Purpose:**

Septic cavernous sinus thrombosis (CST) is a rare complication of infections in the head and neck area. CST is notorious for its bad prognosis, with high mortality and morbidity rates described in literature. However, these rates are based on old series. We question whether the prognosis of CST is currently still as devastating. The primary purpose of this study is to assess the mortality and morbidity of CST.

**Methods:**

Using the databases of all relevant specialties in our tertiary referral hospital, we collected all the patients treated for CST in the period 2005–2017. In addition, a PubMed search, using the mesh term ‘cavernous sinus thrombosis’, was performed.

**Results:**

We found 12 patients with CST in the study period. Of the 12 patients, 11 survived and 9 recovered without any permanent deficits. Seven patients were treated with anticoagulation, and in none of the patients we saw hemorrhagic complications. In literature, older articles describe higher mortality rates (14–80%), but more recent articles report mortality and morbidity rates similar to our results.

**Conclusions:**

The prognosis of CST nowadays is more favorable than previously described. Anticoagulation seems to be a safe addition to antibiotic and surgical treatment, at least in patients without central nervous system infection.

## Introduction

Septic cavernous sinus thrombosis (CST) is known as a rare, life-threatening complication of infections in the head and neck area. Although the use of antibiotics has improved the prognosis, CST is still notorious for its high mortality and morbidity rates. The paranasal sinuses are the most common origin of CST, but also other infections in the head and neck area can cause CST.

Here we present 12 cases of CST of varying origins and discuss the clinical presentation, treatment and outcomes with a multidisciplinary scope. The purpose of this study is to study the mortality and morbidity of CST in our tertiary referral center and to evaluate the effect of treatment with anticoagulants. We discuss our findings within the context of the past and current literature.

## Methods

### Ethical considerations

Formal ethical approval was not required because the preexisting data had been anonymized. All investigations and treatments were carried out in line with accepted clinical practice.

### Study design

We performed a single-center retrospective chart review of patients diagnosed with septic cavernous sinus thrombosis from 01.01.2005 to 31.10.2017. Patients were identified by the databases of the departments of ophthalmology, neurology and otorhinolaryngology. The neurology department has kept a list of all thrombosis patients, of whom we have selected the patients with septic cavernous sinus thrombosis. Both departments of otorhinolaryngology and ophthalmology had lists of admitted patients which included diagnoses, in which cavernous sinus thrombosis could be selected. Both children and adults were included. Data were extracted from the electronic medical records (EPIC). Clinical presentation, imaging, microbiology, surgical operative reports and final outcomes were recorded and analyzed. All imaging (CT and MRI studies) was reassessed by a neuroradiologist.

In addition, a PubMed search, using the mesh term ‘cavernous sinus thrombosis’, was performed. The search was limited to the English language and focused on dated as well as more recent articles.

## Results

### Patient characteristics

Twelve patients were admitted with CST in our institute in the study period. The mean age at diagnosis was 33 years (range 2–79 years). Two patients had a history of diabetes, none of the patients were immunocompromised. A summary of the patients’ characteristics and clinical findings is shown in Table 1.

**Table 1:** Patient characteristics of 12 patients with cavernous sinus thrombosis, listed by age. Abbreviations: CST: cavernous sinus thrombosis; ICA: internal carotid artery; ICU: intensive care unit; LP: lumbar puncture

#	Age	Etiology	Imaging	Lumbar puncture	Microbiology	Antibiotic	Anticoagulation	Surgical intervention	Hospital stay	Follow up imaging (outpatient clinic)	Follow-up/outcome
1	2	Varicella infection	MRI: bilateral CST, bilateral arteriitis and stenosis of the ICA	LP: elevated white blood cel count	Negative	Ceftriaxone, Flucloxacillin	Fraxiparin, 1 month (discontinued after MRI confirmed resolution of thrombosis)	x	13 days (4 days ICU)	MRI after 1 month: normal aspect of bilateral cavernous sinuses, normal flow through bilateral ICA	Follow-up 5 months; complete recovery
2	8	Otitis media/ Mastoiditis	MRI: mastoiditis with thrombophlebitis of the left jugular vein, sigmoid sinus, cavernous sinus and superior ophthalmic vein. Arteriitis of the left ICA	LP: no elevated white blood cell count	*Fusobacterium necrophorum*	Ceftriaxone, Metronidazole, Ofloxacin eardrops	Fraxiparin, Acenocoumarol 3months (stopped without additional imaging)	Mastoidectomy and middle ear drainage	14 days (1 day ICU)	None	Follow-up 12 months: Complete recovery
3	12	Retropharyngeal abscess	CT: Retropharyngeal abscess and bilateral CST	x	*S. Aureus*	Flucloxacillin, Metronidazole	Fraxiparin, Acenocoumarol 6 months (continued after 3 months based on additional MRI)	Drainage of abscess in anesthesia	21 days (3 days ICU)	MRI after 3 months: complete recanalisation of internal jugular vein, residual thrombus in right cavernous sinus	Follow up 12 months: Complete recovery
4	16	Sphenoidal sinusitis	MRI: bilateral CST, bilateral infiltration around the ICA	x	*Gram positive cocci*	Amoxicillin/ clavulanic acid	None	Infundibulotomy, sphenoidotomy	4 days	None	Follow-up 1 month: Complete recovery
5	19	Acute invasive rhinosinusitis ehtmoid sinus	MRI: CST on the right side	x	*Rhizopus oryzae*	Amphoceticin-B, Amoxicillin/ clavulanic acid	None	Draf 3, orbital (eyelid sparing) exenteration	6 days	MRI after 12 months: normal aspect of bilateral cavernous sinuses	Follow up 18 months: orbital reconstruction
6	19	Parapharyngeal abscess	MRI: parapharyngeal abscess collections, right sided CST, arteriitis and stenosis of right ICA, dilated sup. ophthalmic vein	LP: elevated white blood cel count	*Fusobacterium necrophorum*	Ceftriaxone, Metronidazole	Fraxiparin, 3months (discontinued without additional imaging)	Tonsillectomy and abscess drainage	12 days (1 day ICU)	CT after 4 months: normal aspect of bilateral cavernous sinuses and ICA	Follow up 6 months: complete recovery
7	19	Sphenoidal sinusitis	MRI: sphenoidal sinusitis, bilateral CST and stenosis of the ICA	x	*Fusobacterium necrophorum*	Ceftriaxone, Metronidazole	Fraxiparin, Acenocoumarol 3months (discontinued without additional imaging)	Infundibulotomy, sphenoidotomy	25 days	None	Follow up 10 months: complete recovery
8	53	Otitis media/ Mastoiditis	CT: bilateral thrombosis of the confluens, sigmoid sinus, jugular vein, superior ophthalmic vein and cavernous sinus	LP: no elevated white blood cell count	*S.Milleri*	Ceftriaxone, Metronidazole, Flucloxacillin	Fraxiparin, Edoxaban 3months (stopped without additional imaging)	Mastoidectomy, opening of the sigmoid sinus. Liquor drain to reduce liquor pressure	34 days (4 days ICU)	MRI after 6 months: improved flow, only persisting occlusion of left sigmoid and transverse sinus	Follow up 6 months; conductive hearing loss, neurologic and ophthalmic recovery complete
9	53	Sphenoidal sinusitis	MRI: sphenoidal sinusitis with dehiscence of the posterior wall, bilateral CST and left sup. ophthalmic vein thrombosis	x	Negative	Amoxicillin/ clavulanic acid	Fraxiparin, Acenocoumarol (duration 3 months, stopped without additional imaging)	Infundibulotomy, sphenoidotomy	7 days	None	Follow up 2 months: complete recovery
10	56	Sphenoidal sinusitis	MRI: sphenoidal sinusitis with dehiscence and osteitis of the posterior wall. bilateral CST	LP no elevated white blood cell count	*Pneumococcus pneumoniae*	Penicillin	None	Infundibulotomy, sphenoidotomy	9 days	MRI after 2 months: normalising aspectect of cavernous sinus, some residual enhancement	Follow up 3 months: complete recovery
11	61	Sphenoidal sinusitis	MRI: bilateral CST and superior ophthalmic vein thrombosis, sphenoidal sinusitis	LP elevated white blood cell count	Negative	Ceftriaxone, Metronidazole	None	Infundibulotomy, sphenoidotomy	4 days, than out placement	None	Follow up 3 months: complete recovery
12	79	Sphenoidal sinusitis	MRI: left CST and superior ophthalmic vein thrombosis. Subdural empyema, encefalitis	x	*S. Milleri*	Meropenem	None	Infundibulotomy, sphenoidotomy. Drainage of subdural empyema	9 days	None	Died

### Clinical signs and symptoms

The most common clinical symptoms were orbital signs such as eyelid swelling, chemosis, proptosis, impaired ocular motility and reduced visual acuity. In 6 patients ocular motility was impaired in all directions. In 5 patients, only abduction was limited. In one patient, ocular motility was completely normal. Other presenting signs and symptoms were related to the source of infection and included sore throat, trismus, otalgia, and headache. Further neurological deficits were seen in four patients: drowsiness, aphasia, and dysarthria (in two patients).

### Source of infection and micro-organisms

In most patients, CST was caused by sinusitis of the sphenoid sinus (*n* = 6). Other causes were otitis media (*n* = 2), acute invasive fungal rhinosinusitis (*n* = 1), infections in the pharynx (one parapharyngeal and one retropharyngeal abscess), and varicella meningitis (*n* = 1).

The cultured pathogens were very varied: *Fusobacterium necrophorum* (*n* = 3), *S. Milleri* (*n* = 2), *S. Areus* (*n* = 1), *Pneumococcus pneumoniae* (*n* = 1), fungus (*Rhizopus species, n* = 1). Multiple pathogens were cultured in none of the patients. In 4 patients all cultures remained negative.

A diagnostic lumbar puncture (LP) was performed in 6 patients to culture cerebrospinal fluid; in 3 patients a meningitis was confirmed based on the outcome of the LP.

### Imaging

**Fig. 1:**
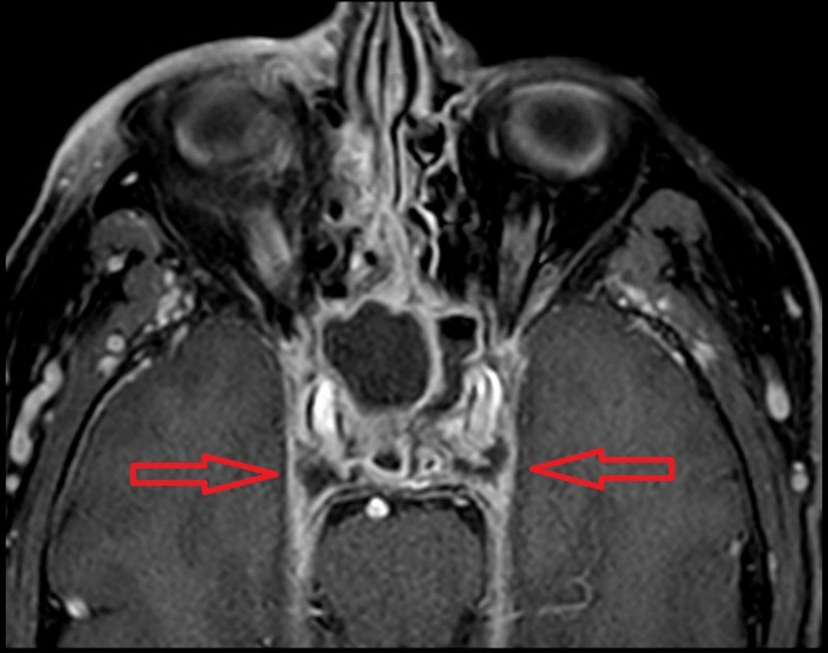
Contrast-enhanced MRI (3D T1GE; E-THRIVE); bilateral filling defect in the cavernous sinus (red arrows) and bilateral opacification of the sphenoid sinus

In all patients, CST was confirmed by imaging, as shown in Fig. 1. Depending on the underlying pathology, availability, and patient characteristics, imaging consisted of a contrast-enhanced CT and/or MRI scan.

**Fig. 2:**
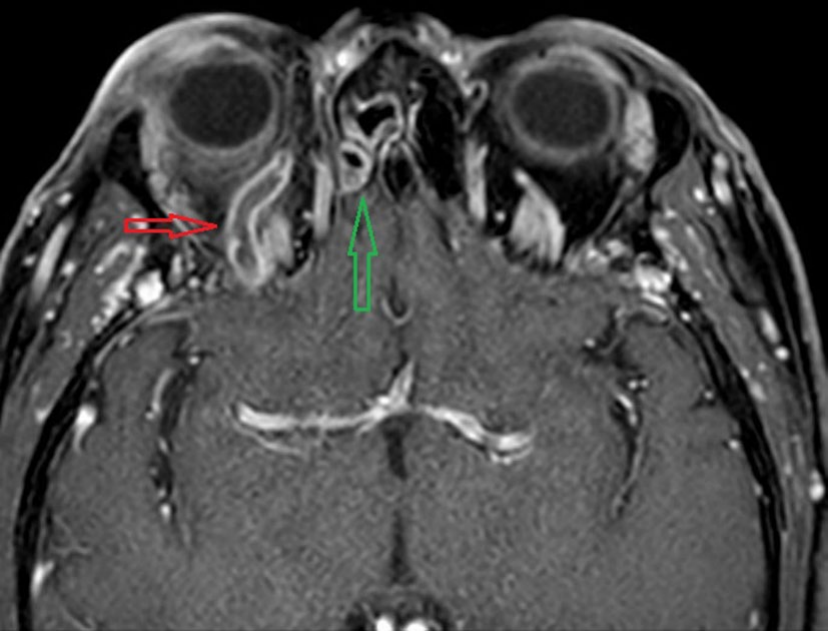
Contrast-enhanced MRI (3D T1GE; E-THRIVE) dilated right superior ophthalmic vein with thrombosis (red arrow) and the enhancement in the right ethmoid sinus (green arrow)

In 10 patients, 15 additional radiological diagnoses were found. Other radiological findings included: thrombosis in other venous sinuses and cerebral veins (*n* = 2), abnormalities (stenosis, arteriitis) of the internal carotid artery (*n* = 5), stenosis and dilatation of the superior ophthalmic vein (Fig. 2) (*n* = 6), subdural empyema (*n* = 1) and cerebritis (*n* = 1).

### Treatment

All patients were treated with antibiotics. The choice of antibiotics was dependent on the (expected) causative micro-organism. The duration of antibiotic treatment was dependent on underlying condition and was always discussed with the microbiologist.

Seven patients received anticoagulation therapy (AT) and 5 patients did not. The decision whether or not to start with anticoagulation therapy was discussed with a neurologist in every case. Meningitis was the reason not to start anticoagulation in three patients in this series. Other reasons to refrain for AT in this series were: small chance of further thrombosis in one patient, high chance of septic embolism in one patient. The duration of AT varied from 1 to 6 months. Most patients (5/7) received AT for 3 months. In one patient AT was stopped after follow-up MRI confirmed resolution of the thrombus, the other patients stopped AT without additional imaging.

Surgery was performed in 11 patients. In most cases (*n* = 7) functional endoscopic sinus surgery (FESS) was performed. The affected sinus was drained and cultures were taken. Other surgical interventions were: tonsillectomy, drainage of a retropharyngeal abscess, orbital (eyelid sparing) exenteration, mastoidectomy and neurosurgical drainage of intracranial empyema (which was performed in the same session as drainage of the sphenoid sinus).

### Outcomes

Eleven out of 12 patients survived, 1 patient died. The patient who died was in a very poor clinical condition upon admission in our hospital. A fulminant cerebritis was the cause of death.

Of the 11 patients who survived the infection and thrombosis, 9 patients recovered without any permanent deficits. The patient with mastoiditis and extensive venous thrombosis recovered with a conductive hearing loss. However, since no preoperative audiometry was available it remains unknown if this hearing loss was preexistent or due to the disease or surgery. The patient with invasive fungal rhinosinusitis of the ethmoid and orbit was completely cured, but orbital exenteration was required for complete removal of the disease. Later, a reconstruction of the soft tissues of the orbit has been carried out using a lateral upper arm flap. There were no bleeding complications in patients treated with anticoagulants.

## Discussion

CST is a rare disorder, although the exact incidence is unclear. Most papers dealing with CST are based on case reports and small single-center series. Cerebral venous thrombosis has an estimated incidence of 13 cases per 1,000,000 per year [1]. The cavernous sinus is the least common location of cerebral venous thrombosis [2, 3]. CST mostly occurs as a complication of sinusitis. Chandler considered CST as the last and most severe complication of orbital cellulitis. [4]. However, other authors consider CST an intracranial complication of sinusitis instead of the end stage of an orbital complication [5–7]. CST is most frequently seen in patients with an infection of the sphenoid sinus, whereas orbital infections are more frequently caused by ethmoiditis [7–9].

In our series, sinusitis is the most common cause of CST (50% of the cases). This is in corroboration with other reports.CST can also be caused by other infections in the head and neck area. Infections of the middle ear and mastoid, mid-facial infections, oral and dental infections, tonsillitis and pharyngitis have been described as sources of CST [9–11]. Distant sources of CST, for example, anorectal abscess, have been mentioned in a few cases [9].

In our series, 33% of cases were due to infections of the head and neck area. That incidence is in agreement with the incidence reported by Weerasinghe et al. [9–<]. We had no distant sources of CST in our series.

### Clinical signs

The most common presenting signs are orbital symptoms (proptosis, chemosis, impaired ocular motility) and cranial nerve impairment [12]. All cranial nerves passing through the cavernous sinus can be affected (III, IV, V1, V2, VI). In our series, the VIth cranial nerve is most frequently impaired. This can be explained by the fact that the VIth nerve runs medially through the sinus, while the other nerves pass through the lateral wall of the cavernous sinus, protected by a thick layer of dura [11–13]. Symptoms of fever and headache also occur in the majority of patients [14].

### Differential diagnosis

CST can be difficult to distinguish from orbital cellulitis. Both can cause eyelid swelling, chemosis, proptosis, double vision, and reduced visual acuity. In orbital cellulitis, the symptoms are usually unilateral. In CST, there is often a unilateral onset with the development of bilateral complaints and symptoms in later stages. Additionally, abnormal pupil responses and papilledema are seen more frequently in cases of CST. Orbital cellulitis usually arises as a complication of paranasal sinusitis, whereas CST can be a complication from any infection in the head and neck area.

Other causes can mimic the symptoms of CST, including tumors, idiopathic orbital inflammation, Tolosa–Hunt Syndrome (inflammatory disease characterized by severe and unilateral headaches with orbital pain and ophthalmoplegia), internal carotid artery aneurysm and carotid-cavernous fistula [15, 16]. However, differentiation with septic CST is normally clear due to the absence of septic signs (such as fever) and the subacute or chronic course.

### Imaging

The diagnosis of CST is made by imaging. We used CT in 3 cases, MRI in 6, and the combination of both in 3 patients. Both direct and indirect signs on CT and MR can be seen in cases of CST. Direct signs are expansion of and filling defects in the cavernous sinus. Indirect signs, caused by venous obstruction, are dilatation of the superior ophthalmic vein, exophthalmos, and increased dural enhancement of the border of the cavernous sinus [17]. Associated findings are secondary thrombosis (for example of the superior ophthalmic vein, petrosal sinus, sphenoparietal sinus and sigmoid sinus) and narrowing of the internal carotid artery [17, 18]. These findings were also frequently found in our series. Besides narrowing of the internal carotid artery, occlusion and aneurysmal formation are also described [19]. We did not find these complications in our series.

Up to date literature about imaging modalities for CST is scarce. Contrast-enhanced CT and MRI are both used to diagnose CST. Eustis et al. reviewed all orbital complications of acute rhinosinusitis, including CST. They prefer MR imaging over CT for CST and describe that false negative CT scans are more common until late in the course of the disease. Their only reference, however, is a single case report which describes an early diagnosis of CST assessed by MR imaging [20].

Schuknecht et al. reviewed 8 patients with CST and compared CT and MR images [17]. They preferred contrast-enhanced CT over contrast-enhanced MR and use CT as primary imaging technique [17].

For cerebral venous thrombosis, T2 weighted MR with gradient echo (GRE) sequence (a sequence more sensitive to tissue susceptibility differences than regular T1 and T2 sequences) is found to be most sensitive for detection of thrombosis. However, in these series of 11, 17, and 45 patients, no patients with CST are included [21–23].

We prefer contrast-enhanced MR imaging to diagnose CST since we found the imaging quality superior to CT imaging in patients with both modalities performed. In addition, potential other intracranial complications can be assessed on MR accurately as well. However, based on the data of this series we cannot draw any conclusion about the sensitivity of MR versus CT imaging.

### Treatment

The main component of the management of CST is treatment of the underlying infection with adequate intravenous antibiotics. Patients in our series received a cephalosporin or penicillin combined with metronidazole, to cover *Staphlycoccus* and *Streptococcus* species as well as Gram-negative and anaerobic species which are frequently involved [15].

Antifungal therapy is indicated in patients at risk for fungal infection; for example, immunocompromised patients or patients with poorly controlled diabetes mellitus.

Surgical treatment also focuses on the underlying infection, and may consist of drainage of sinus, mastoid, pharynx, etc., depending on the source of the infection.

In the last decades, AT is recommended for patients with CST by many authors [9, 11, 15, 24]. One should bear in mind however, that this recommendation is based on retrospective series and reviews, in the absence of clinical trials addressing this subject. In addition, many authors refer to older literature, namely Southwick [14] and Levine [25].

Southwick et al. reviewed case reports of patients with CST treated between 1940 and 1984. Of 86 reported cases, 32% were treated with heparin. Mortality was lower among patients treated with heparin. Of the 8 cases they treated in their own hospital, one received AT. This patient developed a large intracranial hemorrhage due to an overdose of anticoagulants [14].

Levine et al. reviewed their seven cases and literature cases between 1941 and 1988, and found no conclusive evidence for reduced mortality in patients treated with anticoagulation, but they did find a reduced morbidity [25].

In a more recent review of Weerashinge et al. from 2016, 47% of the 88 cases were treated with anticoagulation. The treatment varied in terms of medication and in duration of treatment. Comparing patients with and without anticoagulation, patients treated with anticoagulants had more chance on a full recovery and fewer patients died [9].

Trials performed to investigate if anticoagulation improves mortality and morbidity have been done in patients with cerebral venous thrombosis. In none of these trials, patients had septic thrombosis, let alone CST [26]. In our center, we treat CST with anticoagulants, based on a meta-analysis of two trials that showed a clear benefit of anticoagulants without bleeding complications [26]. We thus have generalized the results of the trials about cerebral venous thrombosis for CST. Although there are differences between these entities, there is considerable overlap in pathophysiology. We, therefore, use data on cerebral venous thrombosis in our considerations for the treatment of CST, as suggested by Desa et al. [27] In patients with meningitis, we usually refrain from anticoagulation, given the increased risk of intracerebral bleeding [28]. The decision whether or not to start with AT is preceded by consultation with a neurologist in every case. The optimal duration of AT is not known, but we normally treat patients for with anticoagulation for 3–6 months, which is a usual term internationally [29].

### Prognosis

In our series of twelve patients, 1 died and 11 survived, of whom 9 without complications. CST has always been notorious for its bad prognosis. Most articles still refer to Southwick, describing a mortality of 50% of their 8 cases and 29% in their literature review of cases treated between 1940 and 1984 [14]. Yarington published a literature review of 878 patients (treated between 1821 and 1960), with a mortality of 80% and a morbidity of 75% [30]. Later, the same author published a series of 28 patients, with a mortality of 13.6% and morbidity of 22.7% [31]. These patients were treated up to 1977. Since then, the mortality rate in many articles has been described as 20–30% in the antibiotic era [11, 12, 15].

For more recent data we have a comprehensive review of case reports, with a mortality of 10/88 (11%) [9]. This review also reveals a high morbidity; of the 78 patients who survived 12/78 (15%) have residual symptoms, of which 8 patients had permanent blindness, 1 patient had double vision, 2 patients had hemiparesis, 1 patient had VIth nerve palsy. In a large multi-center study on cerebral venous thrombosis, the mortality is 8%, however, only a small proportion of the 624 patients had CST [3]. A review of cerebral venous thrombosis describes a mortality of 0–28% [32]. A higher mortality rate (> 5%) was associated with poor clinical condition upon admission (coma, cranial nerve deficits and seizures). The explanation of the decline in mortality of CST in the last decades is sought in improved treatment and identification of less serious case by better quality and availability of imaging techniques. Our study thus adds another contemporary series that shows that mortality is not so dramatically anymore, like several recent articles suggested [24, 33, 34]. Lizé et al. performed a retrospective study of patients with CST as a complication of sinusitis and reported no deaths among their seven patients [24]. Smith et al. published a case series of twelve children with CST and a literature review of pediatric cases. They report a mortality of 0% in their own series and 8% in pediatric cases published between 1994 and 2014. Morbidity rates were 25% in both their own patients and in the selected literature [33]. Wang et al. published a case report and literature review on CST caused by sinusitis between 2002 and 2015. They found a mortality rate of 0% and a morbidity rate of 37.5% [34]. Whether or not AT plays a role in the outcomes of CST is hard to assess by our small series. In the 7 patients treated with AT, we found complete or near complete recovery. However, 3 out of 5 patients not treated with anticoagulation also completely recovered. The one patient who died in the group not treated with anticoagulants was in very bad condition and can be regarded as an outlier. Since we did not find any hemorrhage complications of AT, and considering the findings in literature, we do favor AT in absence of contra-indications.

## Conclusion

Our series and other recent series in literature demonstrate that the prognosis of CST is not as devastating as it previously had been described. Apart from (sphenoidal) sinusitis, other infections in the head and neck area can also cause CST. Cavernous sinus thrombosis can be hard to distinguish from OC, and does not necessarily have a distinct clinical presentation. When imaging is performed in patients with symptoms suspicious for OC, it should be suitable to assess the cavernous sinus, especially in patients with sphenoidal sinusitis. Although to date no consensus about the indications, contra-indications or duration of anticoagulation exist, we advocate AT in patients without contra-indications to their use.
